# The risk of miscarriage is associated with ambient temperature: evidence from coastal Bangladesh

**DOI:** 10.3389/fpubh.2023.1238275

**Published:** 2023-11-03

**Authors:** Susmita Das, Sharoardy Sagar, Srizan Chowdhury, Konok Akter, Muhammad Zahirul Haq, Syed Manzoor Ahmed Hanifi

**Affiliations:** Health Systems and Population Studies Division, International Centre for Diarrhoeal Disease Research, Bangladesh (icddr,b), Dhaka, Bangladesh

**Keywords:** miscarriage, temperature, pregnancy, HDSS, Bangladesh

## Abstract

**Background:**

Exposure to high ambient temperature is reported to cause adverse pregnancy outcomes. However, considering myriad temperature and climatic conditions as well as different contextual factors, the paucity of studies from the developing regions impedes the development of a clear understanding of the heat-pregnancy outcome relationship.

**Materials and methods:**

This study was conducted in Chakaria, a coastal region of Bangladesh, where International Centre for Diarrhoeal Disease Research, Bangladesh (icddr,b) administers a health and demographic surveillance system (HDSS). The surveillance workers visit the households every three months as a part of the routine surveillance activity. Between 2012 and 2020, the surveillance workers documented histories of 23,482 pregnancies among 13,376 women and the women were followed up for their pregnancy outcomes. The temperature records were obtained from the Bangladesh Meteorological Department’s weather station at Cox’s Bazar. The dates of pregnancy outcome were linked with the daily average temperature on the day of pregnancy outcome. A logistic regression model was employed to examine the relationship between temperature and the incidence of miscarriage.

**Results:**

Out of 23,482 pregnancy outcomes, 3.7% were induced abortions. Among the remaining 22,624 pregnancy outcomes, 86.2% were live births, 10.7% were miscarriages and 3.1% were stillbirths. Miscarriages peaked between 8–14 weeks of gestation and varied according to temperature. For women exposed to temperatures between 28°C and 32°C, the risk of miscarriage was 25% greater (adjusted OR 1.25, 95% CI 1.07–1.47) compared to those exposed to temperatures from 16°C to 21°C.

**Conclusion:**

The study establishes a connection between miscarriage and high ambient temperatures in a coastal region of Bangladesh. Implementing timely and appropriate adaptation strategies to prevent miscarriages is of paramount importance for a densely populated country like Bangladesh.

## Introduction

The temperature is inevitably on the rise all over the world due to climate change. In Bangladesh, the coastal region is the most vulnerable hotspot in experiencing the compound effects of climate change (1). Analysis of historical climate data suggests that between 1976 to 2008, the average maximum and minimum temperature in Bangladesh had increased at the rate of 0.018°C per year and 0.015°C per year, respectively, (2). Summers are getting hotter and prolonged while winters are becoming warmer and Bangladesh known for its six seasons is losing its unique seasonality (3). The noticeable impact of rising ambient temperature is more pronounced and unevenly distributed among the vulnerable group of the population living in low-resource settings. Pregnant women are considered highly susceptible to climate-induced rising temperatures (4).

Miscarriage and stillbirth are the most common spontaneous pregnancy losses (5). Every year, an estimated 23 million miscarriages occur worldwide (6). In Bangladesh, there is no national database on miscarriages; however, a survey found that 11.2% of ever-married women were experiencing miscarriages (7). The underlying factors linked with miscarriage are multifaceted. Besides the genetic, maternal, and social factors, we cannot overlook the impact of climatic variables such as extreme temperature on the risk of miscarriage. Existing evidence postulates that extreme heat might impact pregnancy outcomes through multiple mechanisms (8). It has been suggested that heat stress caused by rising temperatures is responsible for hormonal changes and the increased circulatory demands of the fetus on the mother may make the mother vulnerable to heat-related illness (9). Hyperthermia is one of the heat-related conditions and its teratogenicity is dependent on the timing, intensity, and duration of the exposure (10). A maternal core temperature of 39°C is hypothesized as a teratogenic threshold (10–12). Such hyperthermia or high body temperature is an animal teratogen, i.e., known to lower the placental ability to provide nutrition and oxygen (13, 14), induce congenital defects during the crucial stages of fetal development (10). Furthermore, oxidative stress is a recognized trigger that can lead to an imbalance in the reactive oxygen species (ROS) (15) and DNA damage which can result in pregnancy loss (16).

A cohort study conducted among pregnant women suggested that extreme temperature results in various physiological changes such as uterine contraction leading to the detachment of the embryo, and reduced blood flow to the placenta due to vasodilation and dehydration (17). Evidence from an expert group reported that heat exposure impacts pregnant women by increasing the synthesis of several heat shock proteins (4). All of these overwhelming physiological changes are found to be associated with congenital malformations, gestational diabetes, pre-eclampsia, low birth weight, and neonatal stress (4, 11, 18–20). Nonetheless, there has been no exploration of the relation between ambient temperature and adverse pregnancy outcomes in the contextual setting of Bangladesh. It’s difficult to extrapolate the existing findings predominantly originating from developed nations to women in resource-limited settings, as they have different social, economic, and meteorological environments (8). The evidence generated so far does not give a period of vulnerability regarding heat exposure in pregnancy and the likelihood of miscarriage (18) which can inform the policy or practice to safeguard vulnerable women. Hence, more evidence from these nations is needed for informing effective adaptation or mitigation efforts (8) as the mechanistic link between environmental exposure of extreme heat and miscarriage is yet to be fully elucidated. This paper aims to generate a hypothesis regarding the association between ambient heat and miscarriage using available historical temperature records and longitudinal data on maternal health. This preliminary finding will pave the way to a more detailed and robust exploration of the link between extreme temperature and early pregnancy losses.

## Materials and methods

### Setting and population

An HDSS is a system of monitoring a population’s health and demographic traits within a clearly defined geographic area. A baseline census is the first step in the process, which is followed by periodical updates of significant demographic events (births, deaths, and migrations) and health-related incidents (21–23). icddr,b runs three HDSS sites in Matlab, Chakaria, and urban slums. Chakaria HDSS started its operation in 1999. Chakaria is one of the 492 upazilas (sub-district) in Bangladesh and falls under the Cox’s Bazar district. It is located between latitudes 21°34′ and 21°55’ North and longitudes 91°54′ and 92°13′ East on the southeastern coast of the Bay of Bengal. The Chakaria HDSS covers a population of 85,000 living in 17,000 households in 49 villages. Surveillance workers visit each household every 3 months to collect information on basic demographic events, including marriages, pregnancies, births, migrations, and deaths. All of this information is collected using a tablet-based web application. Between 2012 and 2020, a total of 13,376 pregnant women were enlisted in the Chakaria HDSS. We followed these pregnant women for their pregnancy outcomes. All pregnant women registered in the Chakaria HDSS between 2012 and 2020 were included in the study. We excluded all induced abortions from the analysis.

### Exposure assessment

**Figure 1:**
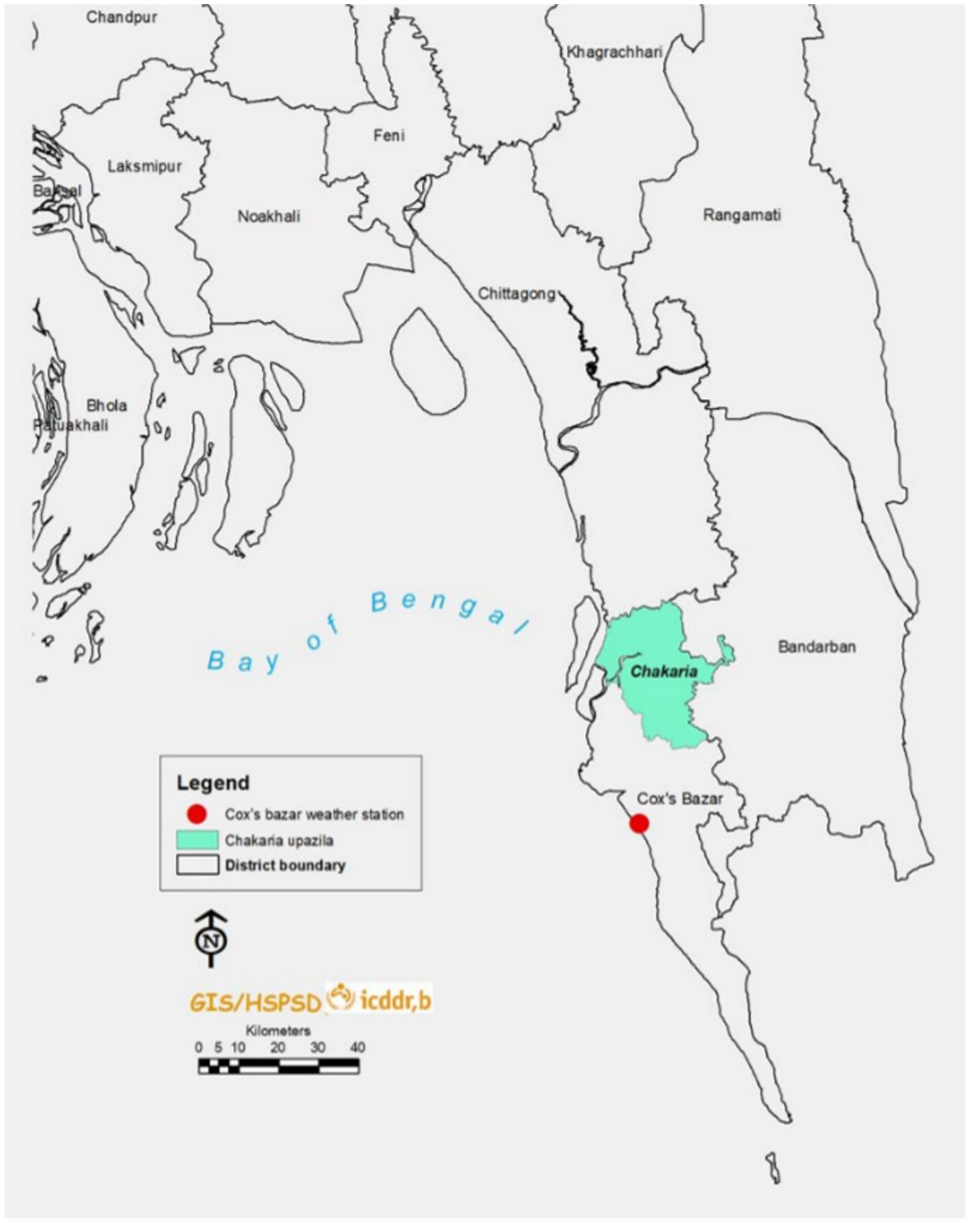
Location of Chakaria (sub-district) and Cox’s Bazar weather station.

The temperature records were taken from Cox’s Bazar weather station of the Bangladesh Meteorological Department (BMD). BMD is the national meteorological organization of Bangladesh that collects meteorological data through weather stations situated across different places in Bangladesh. There is no weather station in Chakaria (sub-district) and hence we have used the temperature records of the Cox’s Bazar (district) weather station (Figure 1). For the exposure (ambient temperature) measurement, the average temperature of the particular day when the pregnancy outcome occurred was considered.

### Outcome assessment

‘Miscarriage’ or spontaneous abortion is usually defined as the loss of pregnancy at less than 20 weeks gestation (24). But miscarriage has been defined differently by different entities, for example, the National Health Service (NHS) defines it as the loss of a pregnancy during the 23 weeks (25) while the American College of Obstetricians and Gynecologists (ACOG) terms miscarriage or early pregnancy loss as the loss of a pregnancy before 13 completed week (26). Therefore, Miscarriages are early pregnancy losses (27–30). For this paper, all pregnancies that got aborted spontaneously before the 28^th^ gestational week were considered as miscarriages. The measurement of gestational age in weeks for this study depends on the data collected through household visits every 3 months as part of the HDSS operation. Women who self-report to be pregnant during the visit are asked to recall the calendar date of the first day of their last menstrual period (LMP). Furthermore, the reports of ultrasound, if done, are checked by the surveillance workers to validate the reported LMP date. The prospective nature of the HDSS data collections, which is open to continuous updates, ensures the accuracy of LMP dates to a reliable degree in contrast with cross-sectional surveys of similar nature. Gestational age based on LMP data collected via rounds of surveillance in Matlab HDSS, another rural HDSS in Bangladesh was found to be highly concordant with that based on the fetus’s crown-rump length by ultrasound during early pregnancy (31), hence HDSS based gestational age can produce valid estimates of adverse pregnancy outcomes (32).

#### Seasons

Four seasons are distinctly recognized in Bangladesh, ‘Winter’ from December to February, ‘Summer’ from March to May, ‘Monsoon’ from June to September, and ‘Autumn’ from October to November (33). We considered these four seasons for this study.

### Distance from the sea

A household’s distance from the sea line is calculated as the aerial distance in meters between the household and the nearest point of the sea line by using ArcGIS software (34), which lies to the left of the HDSS area. The households are located between 8,000 and 24,000 meters from the sea line.

### Statistical analysis

We have included the mother’s age, pregnancy order, education level, and distance from the sea of the mother’s residence (km) as possible covariates. The analysis included the distribution of miscarriages within each category of the exposure and the possible confounders. The mother’s age was calculated from the date of birth as completed years, educational level was collected as years of schooling, and the distance of the mother’s residence from the sea was obtained from the GIS. The average temperature on the day of the pregnancy outcomes was categorized into four groups- 16°C-21°C, (ii) 22°C-24°C, (iii) 25°C-27°C and (iv) 28°C-32°C, mother’s age on the day of pregnancy outcome was categorized as <20 years, 20–24 years, 25–29 years, 30–34 years, ≥ 35 years of age, education level of the mother was categorized as no education, primary education and secondary and above and the distance of the mother’s residence to the sea was categorizes as <20 km and ≥ 20 km. The pregnancy order of the outcome was categorized into four categories- 1, 2–3, 4–5, and 6–8 and the season when the pregnancy outcome occurred was categorized into four categories-summer, monsoon, autumn, and winter.

The Chi-squared test was performed to investigate the association between the outcome and the exposure variables and the significance level of alpha was fixed at 0.05. A logistic regression model was employed to examine the relationship between temperature, the incidence of miscarriage, and the possible confounders. We calculated the crude and adjusted odds ratios (OR) with 95% confidence intervals (CI) to investigate the association.

Survival analysis was performed considering the follow-up time between LMP and miscarriage or completion of 28 weeks of gestation, whichever occurred first. The pregnancy records, contributing one observation per pregnancy, were the unit of analysis. A time-to-event analysis was conducted to depict the higher rate of miscarriage in pregnancies that were exposed to higher temperatures on the day of the pregnancy outcome. The beginning of the risk period was marked by the LMP date of the pregnant woman and the follow-up time i.e.- pregnancy ended in either a miscarriage, which was the event of interest, or it was censored at live birth or stillbirth. The gestational age in weeks was the underlying timescale. The mean ambient temperature from the BMD database on the day the pregnancy ended for each pregnancy was the only measurement of exposure, and it was categorized. The smoothed hazard estimates over gestational age were constructed separately for each category of exposure. All analysis was performed using the STATA software version 17 (35).

## Results

**Figure 2:**
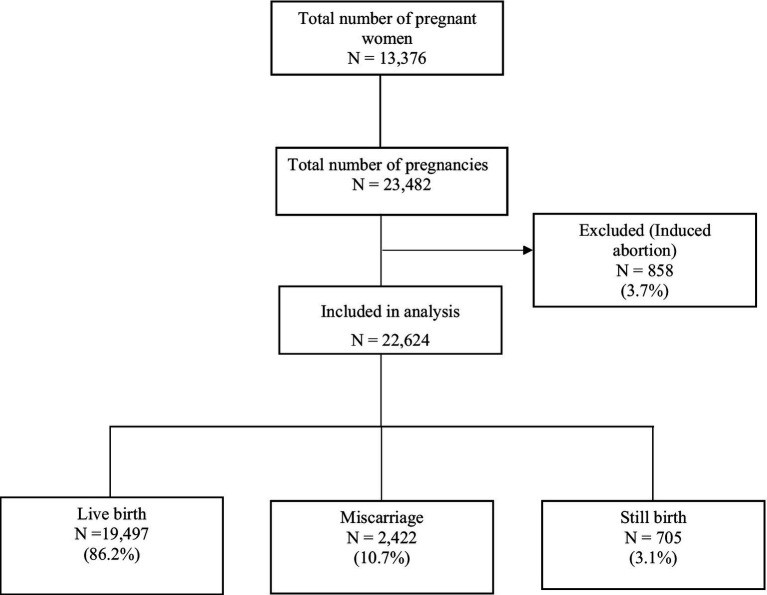
Study population according to inclusion and exclusion criteria (2012–2020).

Between January 2012 and December 2020, 13,376 women were identified and registered in the Chakaria HDSS who were followed for their pregnancy outcomes. There were 23,482 pregnancy outcomes during this period out of which we excluded 858 induced abortions from the analysis. Out of the remaining 22,624 pregnancy outcomes, 19,497 (86.2%) were live births and 2,422 (10.7%) were miscarriages and 705 (3.1%) were stillbirths (Figure 2).

Table 1 describes the pregnancy outcomes by the background demographic characteristics of the respective mothers, the average temperature on the day of the outcome, and the season. More than 70% of the pregnancy outcomes belonged to mothers aged between 20–34 years and 16% of pregnancy outcomes belonged to mothers who were below 20 years. More than half of the pregnancy outcomes were first-order pregnancies. Mothers of nearly one-third of pregnancy outcomes attained education till the primary level and 26% received no education. Mothers of nearly 73% of the pregnancy outcomes resided within 20 km of the Bay of Bengal. The average temperature on the day of the pregnancy outcome was between 28°C-32°C for 44% of the outcomes. More than half of the outcomes took place in Monsoon and Summer.

**Table 1:** Pregnancy outcomes by demographic characteristics, temperature and season.

Characteristics	Proportion (%)
**Age of women (year), *N* = 22,623**	
<20	16.1
20–24	35.8
25–29	26.4
30–34	15.3
≥35	6.4
**Pregnancy order, *N* = 22,624**	
1	57.6
2–3	38.9
4–5	3.4
6–8	0.1
**Educational level of women, *N* = 22,612**	
No education	26.1
Primary	33.1
Secondary or higher	40.8
**Distance from the sea, *N* = 20,232**	
<20 km	72.8
≥20 km	27.2
**Average ambient temperature, *N* = 22,624**	
16°C-21°C	11.0
22°C-24°C	16.3
25°C-27°C	28.5
28°C-32°C	44.2
**Season, *N* = 22,624**	
Summer	23.7
Monsoon	33.4
Autumn	17.6
Winter	25.3

Table 2 shows that miscarriages were more common when the average temperature on the day of pregnancy outcome was between 28°C-32°C and during the summer. Nearly 12% of the miscarriages occurred within 20 km of the coast. Miscarriages were more common among mothers who had no education or who attained primary education. Miscarriage tended to be more frequent among mothers whose age at pregnancy outcome was more than 30 years and higher order births.

**Table 2:** Percentage of miscarriages by demographic characteristics, temperature, and season.

Characteristics	% of miscarriage (Number of miscarriage/Number of pregnancy outcomes)	*p*-value
**Age of women (year)**		
<20	11.1 (404/3,647)	0.000
20–24	9.1 (738/8,093)	
25–29	10.2 (610/5,971)	
30–34	13.1 (454/3,465)	
≥35	14.9 (216/1,447)	
**Pregnancy order**		
1	9.9 (1,288/13,029)	0.000
2–3	11.6 (1,023/8,812)	
4–5	13.9 (106/759)	
6–8	20.8 (5/24)	
**Educational level of women**		
No education	11.2 (660/5,892)	0.048
Primary	11.1 (828/7,489)	
Secondary or higher	10.1 (932/9,231)	
**Distance from the sea**		
<20 km	11.5 (1,693/14,721)	0.000
≥20 km	8.4 (463/5,511)	
**Average ambient temperature**		
16°C-21°C	9.2 (229/2,478)	0.024
22°C-24°C	10.1 (374/3,695)	
25°C-27°C	10.9 (701/6,458)	
28°C-32°C	11.2 (1,118/9,993)	
**Season**		
Summer	12.0 (641/5,355)	0.000
Monsoon	10.9 (825/7,563)	
Autumn	9.4 (374/3,973)	
Winter	10.2 (582/5,733)	

**Figure 3:**
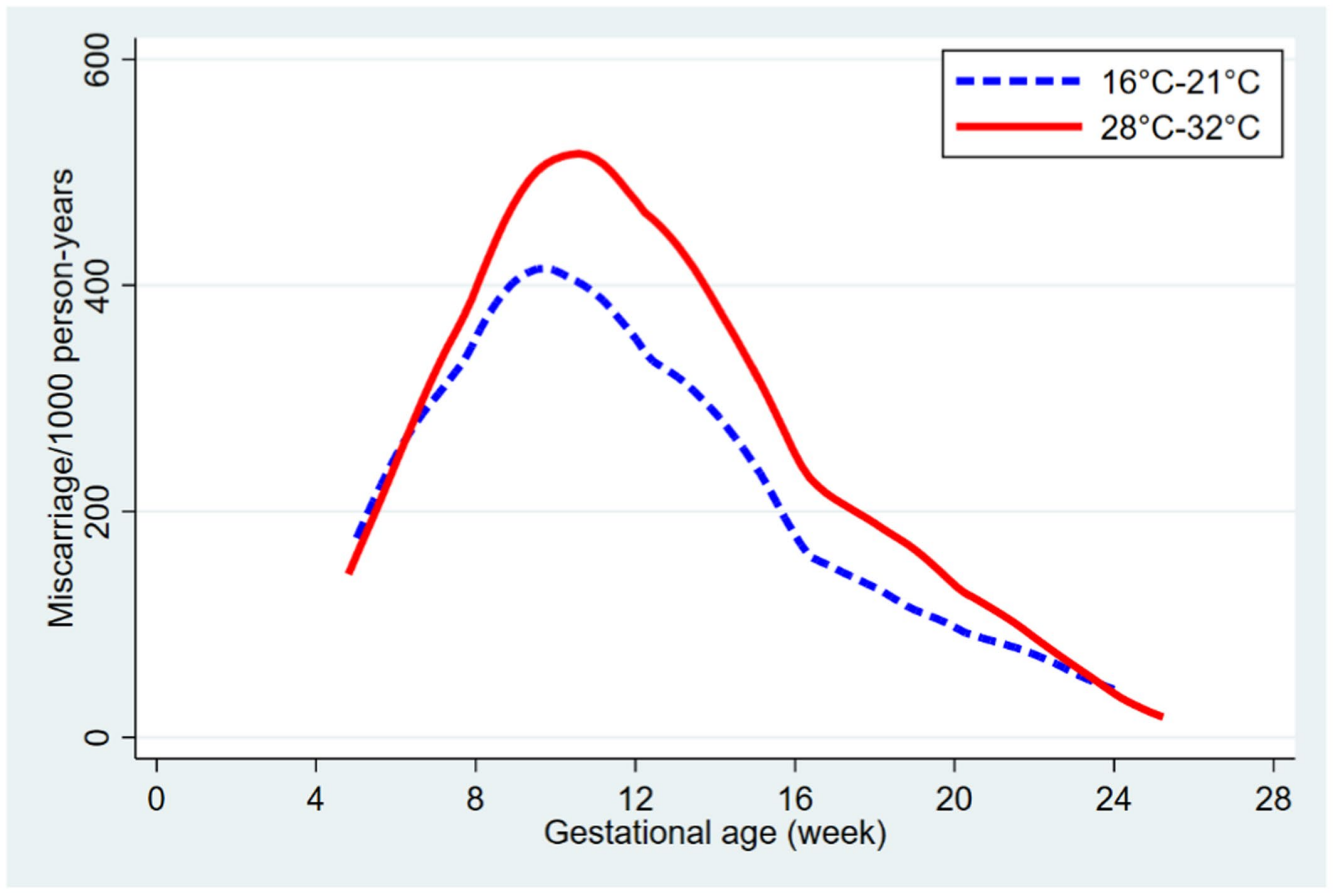
Miscarriage rate per 1,000 person years according to gestational age and temperature.

Figure 3 shows the miscarriage rate per 1,000-person years according to gestational age and across minimum and maximum temperature ranges. The results show that miscarriage rates differed by temperature and higher rates were observed at higher temperatures (28°C-32°C). The miscarriage rate peaked between 8–14 gestational weeks.

Table 3 depicts at temperatures between 28°C-32°C, there was 24% more risk of having miscarriages compared to 16°C-21°C. When adjusted for the mother’s age at pregnancy outcome, pregnancy order, education level, and distance from the sea, the odds for miscarriages at higher temperatures were 25% (95% CI: 1.07–1.47) higher.

**Table 3:** Percentage of miscarriage, crude and adjusted odds ratios according to temperature.

Temperature	% of miscarriage (Number of miscarriage/Number of pregnancy outcomes)	Crude odds ratio (95% CI)	^*^Adjusted odds ratio (95% CI)
16°C-21°C	9.2 (229/2,478)	Ref.	Ref.
22°C-24°C	10.1 (374/3,695)	1.11 (0.93–1.31)	1.13 (0.94–1.36)
25°C-27°C	10.9 (701/6,458)	1.20 (1.02–1.40)	1.22 (1.03–1.45)
28°C-32°C	11.2 (1,118/9,993)	1.24 (1.07–1.44)	1.25 (1.07–1.47)

*Adjusted for mother’s age, pregnancy order, education level and distance from the sea. If any of the information of the possible covariates were missing that sample was excluded from the model.

## Discussion

Our findings support a possible association between high ambient temperature and miscarriages. Miscarriages tended to be more common between 8–14 weeks and among women with lower educational status. It has been reported that about 10 to 15 percent of clinically confirmed pregnancies result in spontaneous miscarriage and the majority happen before 20–28 full weeks of pregnancy, but only 1% of clinically detected pregnancies result in stillbirth (36). While a substantial number of stillbirths can be averted by improving access to quality obstetric care (37), early pregnancy losses can be due to genetic, environmental, social, and behavioral causes and often clinically undetectable (36). Evidence gathered from studies conducted on animals suggests that high maternal temperatures harmed embryonic development processes such as cell proliferation, migration, differentiation, and programmed cell death (apoptosis) which may lead to congenital malformations and embryonic losses (38). However, there are only a handful of explorations on the effect of high ambient temperature on miscarriages. Hajdu and Hajdu’s analysis of more than 30 years of Hungarian administrative data reported that exposure to high temperature increases the rate of spontaneous pregnancy loss which is more pronounced during early pregnancy and among women with low educational status (39). They observed that exposure to high ambient temperature (mean > 25°C) in early pregnancy increased unobserved pregnancy loss rates (36). In a case–control study conducted in Nanjing, Zhao et al. found a non-linear association between high ambient temperature and increased risk of spontaneous abortions (40). Similarly, Sun et al. reported a non-linear association between high ambient temperature and miscarriage (41). Another study from Ghana reported a 15% increase in the likelihood (OR 1.15, 95% CI 0.92–1.42) of having a stillbirth or miscarriage with an increase of each degree temperature (9) but the findings were not statistically significant. Similarly, a study from Turkey did not find any statistically significant relationship between temperature and spontaneous abortion (42) Further, seasonal variation is reported to be associated with spontaneous abortion (43) as well as other types of adverse pregnancy outcomes (44, 45). We observed miscarriages were more common during summer in our study. Likewise, a study conducted in North America reported that the risk of spontaneous abortion was highest during summer (46). However, the association between high ambient temperature and late pregnancy losses (stillbirth) is relatively well investigated. High ambient temperature has been reported to be associated with stillbirths by several studies (47–51). Auger et al. reported that the likelihood of stillbirth from unspecified reasons increased 1.19 times at 28°C compared to 20°C (51), while another study revealed that heat exposure at >23.4°C increased the incidence of stillbirth (52). Chersich and colleagues in their systematic review and meta-analysis reported that the rate of pre-term and stillbirths increases 1.05-fold per 1°C rise in temperature (18). McElroy and colleagues’ analysis of the meteorological and demographic health surveys (DHS) data of 14 LMICs reported that higher maximum temperature in the last week preceding birth increased the risk of pre-term birth and stillbirth and the increased risk starts over 20°C (8). While there are many theories of biological pathways of heat-induced early pregnancy losses there is very little confirmatory evidence. The findings generated from the animal models cannot be completely superimposed for humans as thermal thresholds are strain- and species-specific, and they are specific to developing tissues or organs at particular stages of development (38). As interventional studies to establish the causal relationship between heat and fetal demise are difficult to conduct, more observational studies are needed to fill in the knowledge gap, especially from the global South.

The study is the first of its kind in Bangladesh. It attempted to understand the impact of rising temperatures on pregnancy outcomes with the help of the HDSS platform and the available temperature records in a coastal region of Bangladesh. The findings will support the development and design of contextual intervention programs like shading, cooling facilities, awareness programs, clinical practice and management, and early warning systems to protect vulnerable women. Our study also highlights the need for further investigations to generate a clear understanding of the pathophysiological mechanisms of heat-induced injury, the timing of exposure, and the window of vulnerability (4, 18), which can inform and bring about appropriate policy and interventions to modify the risk posed by rising temperature, especially in the resource-limited settings.

### Strengths and limitations

Our study uses the longitudinal population data from a coastal HDSS with a large sample size, follow-up and comprehensive data, and sub-group analysis that makes the observed relationship more reliable. Also, we use multiple data sources, i.e., population data and available temperature records. This study accounts for the influence of coastal proximity in exploring the link between ambient temperature and miscarriage.

However, our study has several limitations. We have only assessed the effect of the daily average temperature on the day of the pregnancy outcome. Our objective was to generate preliminary evidence on the association using the available temperature records and longitudinal data in the contextual setting of Bangladesh. This evidence shall make way for future studies using more detailed analysis of the ambient temperature for example daily maximum /minimum, lag effects, and other relevant thermal indices. We analyzed the temperature records of the Cox’s Bazar weather station which is nearly 50 kilometers away from Chakaria and may not have correctly reflected local temperatures. The approach we used for statistical analysis also had some limitations. One of the limitations of the method of analysis adopted for this paper is not incorporating the lagged effects of temperature on miscarriage into the model. One such way would be to adopt the framework of distributed lag models (DLM) (20, 53–55), where the outcome is essentially a time series of counts of miscarriage aggregated daily/weekly/monthly. DLM is capable of estimating both linear and non-linear effects of the exposure which would also absolve the need for arbitrarily categorizing the temperature readings before fitting into the model. DLM however would require sufficient counts for the time intervals (daily/weekly/monthly) and has been used for data covering a larger population (53–55). Another possibility of accounting for temperature exposure throughout different parts of the pregnancy period has been explored (41) where the unit analysis remains the pregnant woman and the daily temperature readings are averaged by month or trimester leading up to the day of outcome and plugged into the logistic regression model as covariates using natural or cubic splines. Other variants of the time-to-event analyses or time-series analyses have been employed in estimating the associations between ambient temperature and other adverse pregnancy outcomes e.g.- preterm births (20).

We did not investigate the biological pathway of ambient temperature-related miscarriages. Again, Chakaria is a highly climate-vulnerable, salinity-prone coastal area. Earlier evidence from Chakaria HDSS revealed that miscarriage rates were 1.3 times higher for women living in coastal plains, within 20 kilometers of the coast, and 7 meters above sea level than for those living inland (56). Further, it was reported in another study in Chakaria that housewives consumed more salt than men engaged in labor-intensive occupations (57). Though we tried to adjust for salinity by using distance from the sea as a proxy, there could be other environmental, and socio-cultural factors and maternal infections that may interact with temperature and affect the outcomes. However, recently data loggers have been placed in selected indoor and outdoor locations in Chakaria HDSS which will give real-time temperature readings that can be useful for future explorations.

## Conclusion

To conclude we found a link between high ambient temperature and miscarriages. Keeping in mind the scarcity of evidence from the global south in this regard, it is an important addition to the existing knowledge pool. The findings highlight the need for further exploration especially in the LMIC settings to answer remaining critical queries on this issue that will help formulate appropriate evidence-based policy directives and clinical practices, raise awareness to modify individual behavior, and design adaptation strategies to safeguard pregnant women in the climate-vulnerable resource-limited settings of the world.

## Data availability statement

The raw data supporting the conclusions of this article will be made available by the authors, without undue reservation.

## Ethics statement

The studies involving humans were approved by Institutional Review Board (IRB) of icddr,b. The studies were conducted in accordance with the local legislation and institutional requirements. The participants provided their written informed consent to participate in this study.

## Author contributions

SH and SD conceptualized the paper. SD, KA, SC, and SH prepared the draft manuscript. SS, SC, MH, and SH analyzed the data. All authors contributed to the article and approved the submitted version.

## Abbreviations

ACOG: American College of Obstetricians and Gynecologists
BMD: Bangladesh Meteorological Department
DHS: Demographic health surveys
GIS: Geographic Information System
HDSS: Health and demographic surveillance system
icddr,b: International Centre for Diarrhoeal Disease Research, Bangladesh (icddr,b)
LMIC: Low- and middle-income countries
LMP: Last menstrual period
NHS: National Health Service
ROS: Reactive oxygen species
